# Expression and Interaction Analysis among Saffron ALDHs and Crocetin Dialdehyde

**DOI:** 10.3390/ijms19051409

**Published:** 2018-05-09

**Authors:** Lourdes Gómez-Gómez, Luis F. Pacios, Araceli Diaz-Perales, María Garrido-Arandia, Javier Argandoña, Ángela Rubio-Moraga, Oussama Ahrazem

**Affiliations:** 1 Instituto Botánico, Departamento de Ciencia y Tecnología Agroforestal y Genética, Facultad de Farmacia, Universidad de Castilla-La Mancha, Campus Universitario s/n, 02071 Albacete, Spain; Marialourdes.gomez@uclm.es (L.G.-G.); javier.argandona@uclm.es (J.A.); angela.rubio@uclm.es (A.R.-M.); 2 Centro de Biotecnología y Genómica de Plantas (CBGP, UPM-INIA), Universidad Politécnica de Madrid (UPM)—Instituto Nacional de Investigación y Tecnología Agraria y Alimentaria (INIA), Campus de Montegancedo-UPM, 28223 Pozuelo de Alarcón, Madrid, Spain; luis.fpacios@upm.es (L.F.P.); araceli.diaz@upm.es (A.D.-P.); maria.garrido@upm.es (M.G.-A.); 3 Facultad de Ciencias Ambientales y Bioquímica Toledo, Campus Tecnológico de la Fábrica de Armas, Avda. Carlos III, s/n, E-45071 Toledo, Spain

**Keywords:** saffron, ALDHs, crocetin, structure modelling, docking

## Abstract

In saffron, the cleavage of zeaxanthin by means of CCD2 generates crocetin dialdehyde, which is then converted by an unknown aldehyde dehydrogenase to crocetin. A proteome from saffron stigma was released recently and, based on the expression pattern and correlation analyses, five aldehyde dehydrogenases (ALDHs) were suggested as possible candidates to generate crocetin from crocetin dialdehydes. We selected four of the suggested ALDHs and analyzed their expression in different tissues, determined their activity over crocetin dialdehyde, and performed structure modeling and docking calculation to find their specificity. All the ALDHs were able to convert crocetin dialdehyde to crocetin, but two of them were stigma tissue-specific. Structure modeling and docking analyses revealed that, in all cases, there was a high coverage of residues in the models. All of them showed a very close conformation, indicated by the low root-mean-square deviation (RMSD) values of backbone atoms, which indicate a high similarity among them. However, low affinity between the enzymes and the crocetin dialdehyde were observed. Phylogenetic analysis and binding affinities calculations, including some ALDHs from *Gardenia jasmonoides*, *Crocus sieberi*, and *Buddleja* species that accumulate crocetin and *Bixa orellana* synthetizing the apocarotenoid bixin selected on their expression pattern matching with the accumulation of either crocins or bixin, pointed out that family 2 C4 members might be involved in the conversion of crocetin dialdehyde to crocetin with high specificity.

## 1. Introduction

Saffron spice is produced from the dried stigmas of *Crocus sativus* L., a member of the large Iridaceae family, which has been cultivated in the Near East and the Mediterranean Basin since the Late Bronze Age [1]. The long scarlet stigmas of saffron are highly valued for flavoring and coloring foods and are among the most expensive spices in the world. The compounds responsible for the organoleptic properties of the spice are the apocarotenoids crocins, the glucosylated derivatives of the apocarotenoid crocetin, and picrocrocin, a monoglucosylated derivative of 4-hydroxy-2,6,6-trimethyl-1-cyclohexene-1-carboxaldehyde (HTCC), which gives the stigma its red coloration and provides saffron with its bitter taste, respectively [2]. In recent years, public interest in the use of natural additives as substitutes for synthetic chemicals has increased the use of saffron as a natural flavoring in the food industry, along with increased interest in the biological effects and potential medical applications of saffron [3,4,5]. Clinical trials indicate a beneficial role in the treatment of depression [6] and dementia [7] and as an anticancer agent [8]. Furthermore, saffron acts as a powerful free radical quencher and displays a variety of health benefits, which has led to it being used in traditional medicine in various countries. The accumulation of crocins is not restricted to saffron. Wild crocus varieties such as *C. ancyrensis*, *Gardenia jasminoides* and some species of lamiales do, although in minor quantities [9].

The biosynthesis of carotenoids and apocarotenoids in the saffron stigma begins in the earliest stage of flower development [10]. Saffron stigma produces volatile and non-volatile apocarotenoids in different stages of flower development. Among the non-volatile apocarotenoids crocins, which accumulate at the highest levels in stigmas, are synthesized in the saffron chromoplast through a specialized carotenoid biosynthetic pathway [11,12]. The first step in the carotenoid pathway is catalyzed by phytoene synthase (PSY), resulting in the condensation of two C-20 geranylgeranyl diphosphate (GGPP) molecules to form phytoene [13]. Phytoene desaturase (PDS) and ζ-carotene desaturase (ZDS) catalyze the formation of lycopene from phytoene by adding four double bonds. The desaturation reaction increases the number of conjugated double bonds and occurs sequentially by transforming phytoene into phytofluene, ζ-carotene, neurosporene, and lycopene [14]. Two specific isomerase enzymes are required for the formation of all trans-isomers of lycopene, 15-*cis*-ζ-carotene isomerase (Z-ISO) and the carotenoid isomerase (CRTISO). Cyclation of one or both ends of the linear C-40 hydrocarbon chain of all-trans-lycopene allows the formation of two types of carotenoids: carotenoids with two β rings like β-carotene and derived β,β-xanthophylls such as zeaxanthin; and those that contain one β ring and one ε ring as β-carotene, α-carotene, and derived β,ε-xanthophylls such as lutein [15]. These reactions are catalyzed by lycopene β-cyclase (LCYB) and lycopene ε-cyclase (LCYE) activities, producing β and ε rings, respectively. Carotenoid hydroxylases (CHYs) catalyze the hydroxylation of β and ε rings [16]. Carotenoid cleavage dioxygenase enzymes (CCDs) from different subfamilies 1, 2, 4, 7 use carotenoids as substrates to synthesize apocarotenoids [17,18,19]. The enzyme CCD2, localized in the chromoplast [20], catalyzed the cleavage of the 7,8 and 7′,8′ double bonds of this carotenoid to render one molecule of crocetin dialdehyde and two molecules of 8-hydroxy-β-cyclocitral [21]. The crocetin dialdehyde is further oxidized by an unknown aldehyde dehydrogenase (ALDH) to obtain crocetin, which is later glucosylated [22], generating soluble crocins that accumulate in the vacuole [23]. The biosynthesis pathway of crocins from zeaxanthin is shown in the Supplementary Materials (Figure S1).

The ALDH superfamily is composed of a vast number of enzymes involved in many processes such as carnitine biosynthesis, glycolysis/gluconeogenesis, cellular redox balance maintenance and amino acid metabolism [24,25,26,27]. ALDH enzymes use either Nicotinamide Adenine Dinucleotide (NAD^+^) or Nicotinamide Adenine Dinucleotide Phosphate (NADP^+^) as a cofactor to convert aldehydes to their corresponding carboxylic acids [28]. The number of ALDHs found in plants ranges from seven found in the green algae *Volvox carteri* to 26 in *Populus trichocarpa* [28]. Plant ALDH proteins are found to be localized in the cytosol, mitochondria, plastids (chloroplasts, chromoplasts and leucoplasts), peroxisomes, and microsomes [29,30]. ALDH expression is regulated throughout plant tissues and developmental stages and is altered by abiotic stresses such as dehydration, water logging, high salinity, heavy metals, heat, oxidative stress, cold, ultraviolet radiation, and many others [31,32,33], suggesting a role as aldehyde scavengers under conditions inducing oxidative stress [34].

In the present investigation, we selected ALDH candidates based on the chromoplast proteome obtained from saffron stigmas previously published [23] to determine by means of structure modeling and docking calculations, gene expression, and in vitro activities experiments whether these ALDHs are able to use crocetin dialdehyde to produce crocetin; we also estimated binding affinities of some ALDHs to crocetin dialdehyde from crocetin species producers.

## 2. Results and Discussion

In saffron, the non-volatile apocarotenoid crocetin dialdehyde is obtained by the action of the plastidic enzyme CCD2 [20,21] over the carotenoid zeaxanthin, which is further oxidized by an unknown aldehyde dehydrogenase to obtain crocetin. Recently, Gomez et al. [23] analyzed the chromoplast proteome from saffron stigmas and, based on the expression pattern and correlation analyses, pinpointed five ALDHs as possible candidates to generate crocetin from crocetin dialdehydes, two of them almost identical. Therefore, in this study, we include only four ALDHs: ALDH3898 (accession number KU577905), ALDH20158 (accession number KU577906), ALDH54788 (accession number KU577904), and ALDH11367 (accession number KU577907). The four ALDHs gene sequences coded for members of three ALDH protein families: ALDHs 20158 and 11367 belong to family 2, which includes mitochondrial and cytosolic enzymes with relatively broad substrate specificity [24], ALDH54788 falls into family 3, having members with different subcellular localization including cytosol, chloroplasts and endoplasmic reticulum [35,36,37], while ALDH3898 shows identity with members of family 6, also known as methyltmalonyl semialdehyde deshydrogenase, the only ALDH family able to use coenzyme A (CoA) as a cofactor [28]. It has been reported that families 2, 3, and 6 are present in plants and algae, suggesting that these families have ancient origins that precede the transition of aquatic to terrestrial habitats [9,38].

In mammals, ALDH2 has been reported to play an important role in detoxifying lipid peroxidation-derived aldehydes produced under different stress [33]. In plants, the ALDH2 family is relatively diverse. In rice, the role of ALDH2 was described as a nuclear restorer (rf2) of cytoplasmic male sterility (cms) [39]. Nevertheless, clear functions of both mitochondrial and cytosolic proteins in plants have not been identified. In *Arabidopsis*, tomato, and *Populus*, the families ALDH2 and ALDH3 are composed of many members, which suggest that they are functionally important, performing detoxification of aldehyde molecules generated during oxidative stress and maintaining homeostasis, while only one member of the ALDH6 family has been found in these species [9].

The ALDH proteins have predicted isoelectric points from 6.88 to 8.83 and range from 482 to 538 amino acids in length, with ALDH3898 and ALDH54788 being the largest and shortest proteins, respectively. ScanProsite analysis revealed the presence of the characteristic domains of ALDHs PS00070 (cysteine active site) and PS00687 (glutamic acid active site), which were present in ALDHs 20158 and 11367 whereas ALDH3898 and ALDH54788 showed only PS00070 and PS00687, respectively.

To determine whether these genes are specific to stigma tissue or not, a qPCR analysis was performed in leaf, root, cormlet, and stigmas at the red stage as described in Section 3.4. RNA Extraction and Quantitative Real-Time PCR Analysis. The expression pattern of the ALDHs was different among the three families (Table 1). *ALDH20158* and *ALDH11367* transcripts were not detected in any of the tissues analyzed and were only found in stigmas, with ALDH11367 having the highest expression level in this tissue. *ALDH3898* and *ALDH54788* were detected in all tissues except for cormlet, where no expression was found. The expression peak of the *ALDH3898* and *ALDH54788* was detected in root tissue.

**Figure 1:**
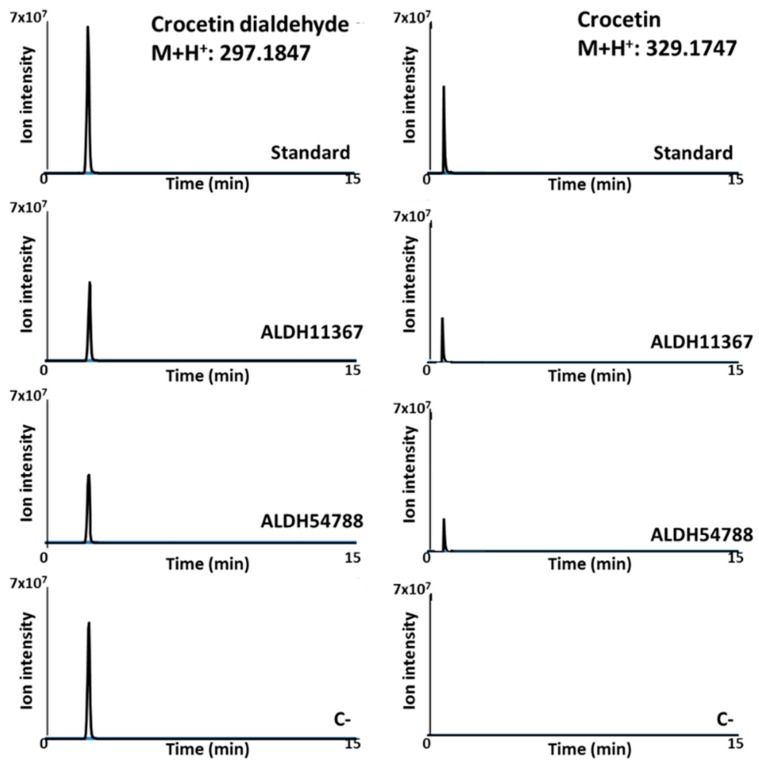
Chromatograms obtained after the analysis by LC-APCI(+)-MS from the enzymatic reactions using crude proteins extracts from *E. coli* cells expressing ALDH11367, ALDH54788, and crocetin dialdehyde as substrate. C-: negative control using crude proteins extracts from *E. coli* cells expressing empty pThio-Dan1.

In order to determine the activity of the four ALDHs, we first cloned these enzymes using In-Fusion technology to yield thioredoxin fusion proteins in the arabinose-inducible vector pThio-DAN1. The recombinant proteins were expressed in *E. coli* strain Bl21(De3). SDS/PAGE analysis showed that the four ALDHs fusions were expressed with the correct apparent molecular mass 53, 57, and 58 kDa. We used an in vitro assay to explore the activities of these enzymes. The enzymatic activity was assayed in vitro with crude protein extracts. As demonstrated by LC-APCI(+)-MS analysis, incubation with crocetin dialdehyde resulted in the formation of crocetin (Figure 1). Only chromatograms from ALDH11367 and ALDH54788 are shown; the same results were obtained using ALDH20158 and ALDH3898.

To gain insights into the biological role played by the ALDHs from saffron, we modeled these enzymes. To construct the model structures of the four ALDHs, initial sequence alignments in the homology modeling process were performed, identifying the following proteins as templates: Human Sjogren–Larsson Syndrome enzyme fatty aldehyde dehydrogenase (PDB id 4QGK) [40] for ALDH54788, methylmalonate-semialdehyde dehydrogenase DddC from *Oceanimonas doudoroffii* (PDB id 4ZZ7) [41] for ALDH3898, and sheep aldehyde dehydrogenase 1A1 (PDB id 5ABM) [42] for both ALDH20158 and ALDH11367. In all cases, template proteins had a reasonably high sequence identity and the superposition between template and model structures revealed a high coverage of residues in the models, showing very close conformations indicated by the low RMSD values of backbone atoms (Table 2).

**Figure 2:**
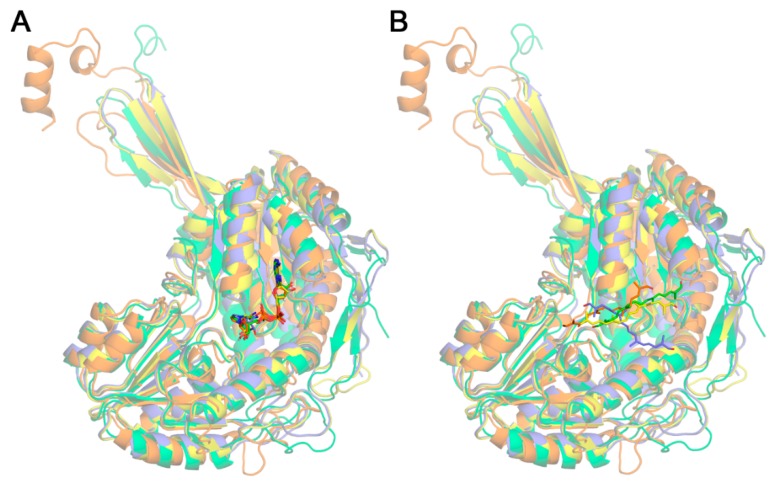
Structural optimized superposition of the four model structures of ALDHs (ribbons) showing the best geometries of (**A**) NAD coenzyme and (**B**) crocetin dialdehyde (sticks). ALDH54788 in orange, ALDH3898 in green, ALDH20158 in yellow, ALDH11367 in slate blue.

As expected, the four models exhibit the ALDH architecture displaying the Rossmann fold motif present in NAD-binding enzymes (Figure 2). However, their structural alignment reveals non-negligible differences. In fact, taking ALDH54788 model as reference, the optimized structural pairwise comparisons give the following results expressed as number of superposed residues/total number of residues in the protein and the corresponding root-mean-square deviation (RMSD) computed with backbone atoms. ALDH3898: 323/488 and 1.459 Å, ALDH20158: 331/485 and 1.506 Å, and ALDH11367: 331/481 and 1.530 Å. The relatively low number of superposed residues (below 70%) together with the relatively high value of RMSDs (~1.5 Å) indicates that the structural models show indeed noticeable differences in spite of sharing the same architecture. This notwithstanding, the geometries of NAD coenzymes in the four models reveal an excellent agreement with nicotinamide rings and adenine moieties showing nearly coincident spatial locations (Figure 2A). Given that the positions of NAD were obtained upon optimizing geometries, this result indicates a high structural conservation of the binding site in the four ALDHs.

**Figure 3:**
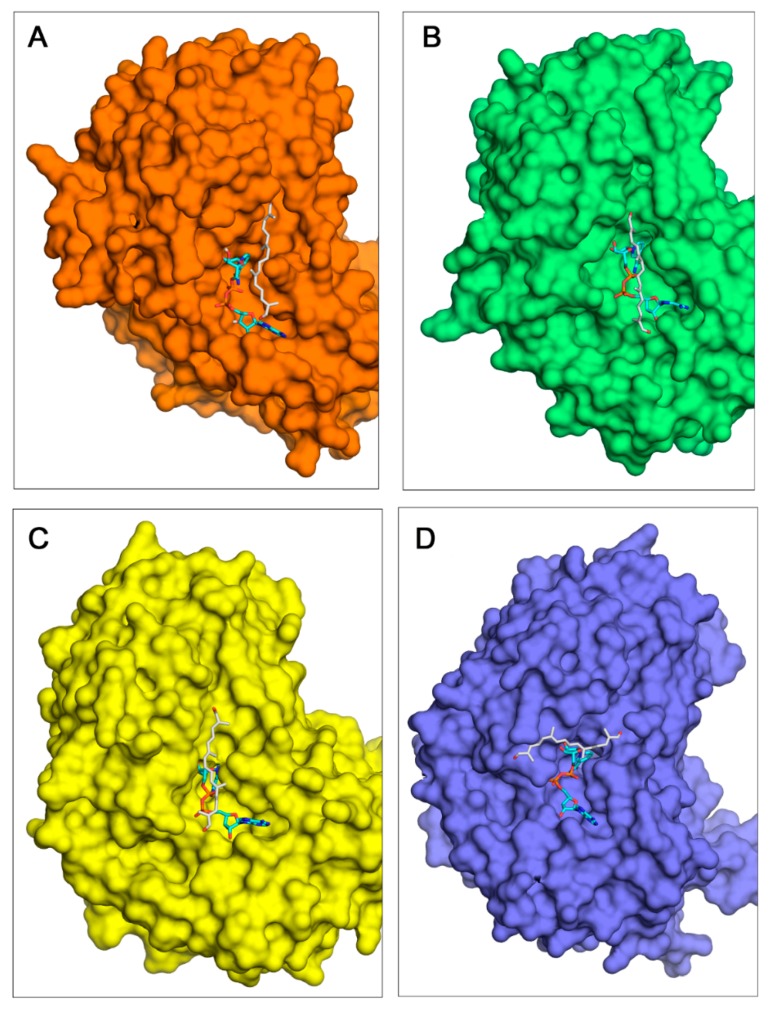
Molecular surfaces of the four model structures of ALDHs showing the best docking geometries of NAD coenzyme (sticks with carbons in cyan) and crocetin dialdehyde (sticks with carbons in light grey). (**A**) ALDH54788; (**B**) ALDH3898; (**C**) ALDH20158; (**D**) ALDH11367.

Docking calculations were performed to obtain the complexes of the four enzymes with crocetin dialdehyde. Despite the close agreement in the spatial location of NAD in the four ALDH models, docked geometries for crocetin dialdehyde reveal significant differences in the particular orientation (Figure 2B). Given that all the docking calculations were performed with exactly the same input parameters and grid definition, this result suggests on the one hand that the abovementioned structural differences should have an effect on the precise orientation of ligands in the binding site of these ALDHs, and on the other hand that the affinity of these ALDHs to crocetin dialdehyde is not very high. In fact, those structural differences do not significantly affect the binding affinities ΔG^0^ estimated in the docking calculations. Not only do the four complexes display rather similar ΔG^0^ values (Table 3), but other ligand geometries obtained in every docking happen to show very small energy differences of about 0.1–0.3 kcal/mol while having distinct 3D locations in the binding site. These protein–ligand ΔG^0^ estimated affinities indicate a weak affinity of ALDHs to crocetin dialdehyde with complex dissociation constants in the high μM range (Table 3). In any event, the molecular surface of the four ALDH–crocetin dialdehyde models revealed a deep pocket for the coenzyme with clear shape complementarity and a large elongated cleft for crocetin dialdehyde (Figure 3). Except in the case of ALDH11367 (Figure 3D), the orientation of crocetin dialdehyde in these complexes is similar.

A detailed analysis of these binding sites suggest that the catalytic activity should need a conformational motion of either crocetin dialdehyde or NAD (or both) to properly locate one of the oxygens of the ligand near the nicotinamide ring of the coenzyme, which participate in the redox process underlying the enzymatic activity and which is deeply buried in the pocket (Figure 2).

The fact that the four ALDHs from *C. sativus* were able to act over crocetin dialdehyde to produce crocetin is not surprising. In 2016, Frusciante et al. [20,21] investigated the role of CCD2 and performed a transient expression in yellow maize endosperm, the analysis of the CCD2-expressing samples by quantitative LC-HRMS revealed the absence of the cleavage intermediate 3-OH-β-apo-8′-carotenal (β-citraurin) and the final product crocetin dialdehyde. However, a new peak with an *m*/*z* of 329.1747 was detected and characterized as crocetin. They also tested *E. coli* strains accumulating zeaxanthin upon expression of CCD2 and two metabolites have been detected, i.e., crocetin dialdehyde and crocetin. The results pointed out that *E. coli* seems to contain an aldehyde dehydrogenase able to convert crocetin dialdehyde to crocetin. Using a rice system suited to the functional characterization of carotenoid biosynthesis genes [43], Ahrazem et al. [20] transformed six-day-old mature zygotic rice embryos with a cocktail of constructs driven by endosperm-specific promoters: ZmPSY1, PaCRTI and AtDXS in combination with CaCCD2, a CsCCD2 homologue from the spring *Crocus ancyrensis*, driven by the maize ubiquitin 1 promoter and intron 1 [44] without co-expressing any aldehyde dehydrogenase, led to the generation of several independent callus lines under hygromycin selection showing different color intensity ranging from white through yellow to orange. Lines expressing ZmPSY1, PaCRTI, AtDXS, and CaCCD2 or only AtDXS and CaCCD2 revealed the presence of crocetin with different amount of accumulation among the lines. Recently, two out of the three CCD4 enzymes, namely BdCCD4.1 and BdCCD4.3 characterized from Buddleja, showed 7,8;7′,8′ activity in vitro and in vivo over zeaxanthin. HPLC analysis of the extracts from the recombinant proteins co-expressed in carotenoid-accumulating *E. coli* strains engineered to accumulate zeaxanthin, via pAC-ZEAX showed again the presence of crocetin extract instead of crocetin dialdehyde, which reinforces the presence of a non-specific aldehyde dehydrogenase from *E. coli* and plants able to produce crocetin [9].

**Figure 4:**
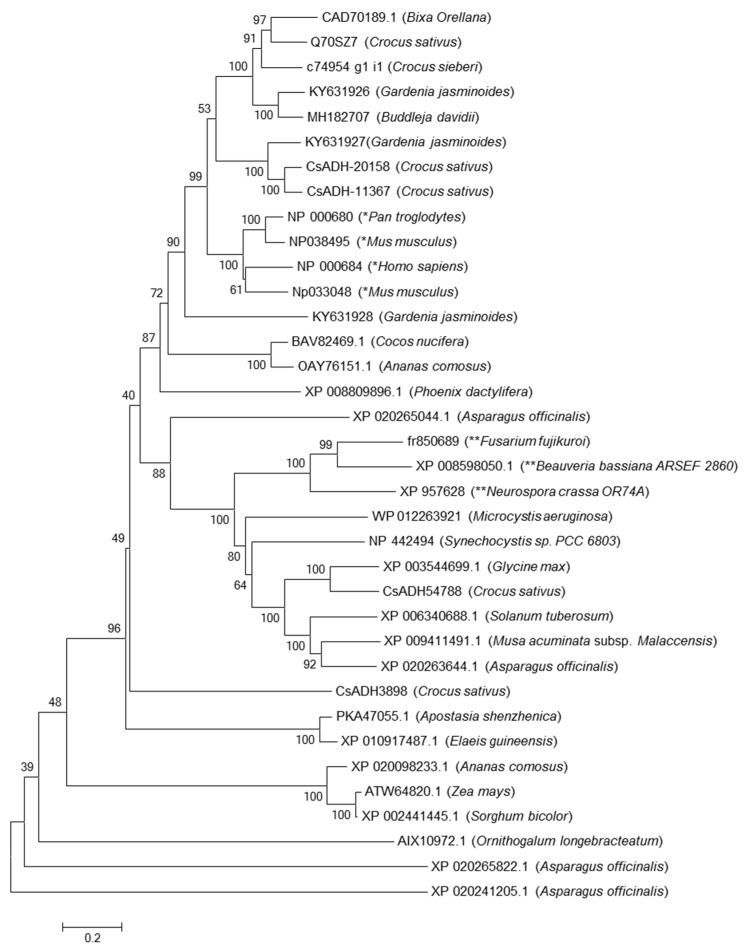
Phylogenetic tree showing relatedness of full-length ADHs from several plant and some fungi species, based on amino acid sequence similarity. Sequences from animals (*) and fungi (**) are highlighted with asterisk. The tree was generated using bootstrap support based on 9000 replicates constructed using MEGA version 6. Numbers at nodes indicate degree of bootstrap support. Branch distance indicates proportion of amino acid changes: a distance of 0.2 is equal to 20 changes per 100 amino acids. The predicted protein sequences used to generate the tree are listed in the Supplementary Materials.

Recently, transcriptomes from *Gardenia jasmonoides*, *Bixa orellana*, *Buddleja davidii*, and *Crocus sieberi* have been published [45,46,47]. We manually looked for different ALDHs genes from these species and selected three from *G. jasminoides* (KY631926, KY631927 and KY631928) and one *B. davidii* (accession number MH182707) and *C. sieberi* (c74954_g1_i1 transcriptome accession PRJNA413953) based on their expression pattern matching with the accumulation of crocins, and one ALDH from *Bixa orellana* (accession number CAD70189.1) species that accumulates the C-24 apocarotenoid bixin, which has been selected using the same criteria. KY631927 and KY631928 belong to family 14 and 18, respectively, while KY631926, CAD70189.1, Q70SZ7, MH182707, and c74954_g1_i1 fall in family 2 C4. We performed an alignment together with other ALDHs and drew a phylogenetic tree to see whether they clustered together with the studied ALDHs or not (Figure 4). The resulting tree was validated as it showed that the fungi ALDHs grouped together and the retinal ALDHs were found to be clustered in the same group. Only, KY631927 from gardenia were clustered with ALDH-20158, and ALDH-11367, on the other hand; the KY631926 from Gardenia CAD70189.1 from Bixa, MH182707 from Buddleja and the c74954_g1_i1 from *C. sieberi* together with Q70SZ7 from *C. sativus* were assembled in the same subgroup and formed a cluster more related to the group of ALDHs involved in the conversion of retinal to retinoic acid together with ALDH KY631928 from gardenia and far away from the ALDHs from fungi that are able to oxidize the aldehyde group of different apocarotenals generating mainly β-apo-4′-carotenoic acid or β-apo-4′-lycopenoic acid (Figure 4). KY631926, c74954_g1_i1, CAD70189.1, MH182707and Q70SZ7 are members of ALDH2 C4, the homologue identified in *Arabidopsis* plays a role in biosynthesis of ferulic acid and sinapic acid, important compounds involved in cell wall strength [48]; however, specific functions for members of family 2 C4 have not been identified yet.

To check the affinity between crocetin dialdehyde and these proteins, binding affinities ΔG^0^ were estimated for all members of family 2 C4, using the same input parameters and grid definition, the results are shown in Table 4. All the ΔG^0^ were higher than those obtained for the ALDHs20158, 11367, 3898 and 54788 from saffron. The highest and lowest were calculated for *Gardenia jasmonoides* KY631926 and *Bixa orellana* CAD70189.1, respectively; the ALDHs Q70SZ7 from saffron showed a ΔG**^0^** of −8.2 kcal/mol. The species accumulating crocetin showed a dissociation constant ranging from 0.2 to 1.3 while *B. orellana*, which accumulates bixin by cleavage of lycopene at both the 5,6 and 5′,6′ positions, has a K_d_ higher than the ALDHs selected from the crocetin accumulating species. These data indicate that these proteins have much more affinity for the crocetin dialdehyde, as revealed by their lower K_d_, and might play a major role in the conversion of crocetin dialdehyde to crocetin.

## 3. Materials and Methods

### 3.1. Cloning of ALDHs

Stigmas at different developmental stages were obtained from *C. sativus* grown under field conditions in the Botanical Garden of CLM (Albacete, Spain). The tissues were frozen in liquid nitrogen and stored at −80 °C until required.

The ALDH gene from *C. sativus* was amplified by PCR from purified cDNA with specific oligonucleotide primers listed in Table 1 based on the sequence of the gene available at NCBI. The PCR products were cloned using In-Fusion^®^ HD Cloning Kit (clonthec, Takara, Japan) according to the manufacturer’s instructions into pTHIO-Dan1 previously cut with EcoRI. *E. coli* JM109 was used for the propagation of recombinant plasmids. The correct sequences of the genes cloned into the vectors were verified by DNA sequencing using an automated DNA sequencer (ABI PRISM 3730xl, Perkin Elmer, Macrogen Inc., Seoul, Korea).

PCR was performed using Advantage high-fidelity DNA polymerase (Clonthec, Takara, Japan) according to the manufacturer’s instructions. PCR fragments were purified from agarose gel bands using Wizard(R) SV Gel and PCR Clean-Up System kit (Promega, Madison, WI, USA).

### 3.2. Recombinant Expression of ALDHs

For protein expression, *E. coli* Bl21(De3) strain were transformed with pThio-Dan1-Aldhs. Then 50 mL of 2× YT medium (Sigma-Aldrich, Madrid, Spain) were inoculated with 3 mL of overnight pre-culture and grown at 28 °C until 0.5–0.8 of absorbance at 600 nm was reached. Then protein expression was induced using 0.2% *w*/*v* arabinose and incubation at 22 °C overnight. Cells were harvested by centrifugation at 5000× *g* for 10 min. The pellet was resuspended in 1 mL lysis buffer (50 mM NaH_2_PO_4_, 300 mM NaCl, 1 mg/mL lysozyme, 1 mM dithiothreitol, 0.1% *v*/*v* Triton X-100, pH 8.0) and incubated on ice for 30 min. After sonication, the lysate was centrifuged for 30 min at 12,000× *g* and the supernatant was transferred to a new tube and used for in vitro assays.

### 3.3. Enzymes Assay and Analysis of Reaction Products

Crocetin dialdehyde was dissolved in 100 μL of incubation buffer (200 mm pyrophosphate, 200 mm NaCl, pH 7.5, 2 μL of 100 mm NAD^+^), and 80 μL of crude protein and water were added to give a total volume of 200 μL. Incubations were performed at 28 °C for 30 min, stopped by addition of 1 mL of acetone, extracted with light petroleum/diethyl ether (1:4, *v*/*v*). The reactions were analyzed by LC-APCI(+)-MS, using a Q-exactive quadrupole Orbitrap mass spectrometry system (ThermoFisher Scientific, Madrid, Spain), coupled to a HPLC system equipped with a photodiode array detector (Dionex, Madrid, Spain) as described [9].

### 3.4. RNA Extraction and Quantitative Real-Time PCR Analysis

Total RNA extractions were performed as reported [49]. The quantitative RT-PCR was carried out on cDNA from three biological replicates; reactions were set up in GoTaq^®^ qPCR Master Mix (Promega, Madison WI, USA) according to manufacturer’s instructions, with gene-specific primers (0.125 µM) in a final volume of 25 µL. The Primer design was performed using Primer3 program (http://frodo.wi.mit.edu/) [50]. Primer sequences are listed in the Supplementary Materials. The constitutive expression gene 18SrRNA was used as the reference gene. The cycling parameters of qPCR consisted of an initial denaturation at 94 °C for 5 min; 40 subsequent cycles of denaturation at 94 °C for 20 s, annealing at 58 °C for 20 s and extension at 72 °C for 20 s; and final extension at 72 °C for 5 min. Assays were conducted with a StepOne™ Thermal Cycler (Applied Biosystems, Foster City, CA, USA) and analyzed using StepOne software v2.0 (Applied Biosystems). Analyses of qRT-PCR data used the classic (1 + E)^−ΔΔ*C*t^ method (*C*_t_ is the threshold cycles of one gene, E is the amplification efficiency). Melt curves were created for each primer combination to confirm the presence of a single product. The qPCR products were separated on a 1.0% agarose gel and, then, were sequenced to confirm their identity using an automated DNA sequencer (ABI PRISM 3730xl, Perkin Elmer) from Macrogen Inc. (Seoul, Korea).

### 3.5. Structure Modelling

Structures of the four aldehyde-dehydrogenases were constructed by homology modeling with Swiss-Model [51,52,53]. In all cases, the model with best quality scores Global Model Quality Estimation (GMQE) and Qualitative Model Energy Analysis (QMEAN4) was selected. Initial structure of NAD coenzyme was taken from the crystal structure of class 3 ALDH from rat (PDB id 1AD3 [54]) because it (i) includes the coordinates of NAD, (ii) has high sequence and structural similarity with the four ALDHs modeled in this work, and (iii) is not selected as template for any of them. Each ALDH model structure was superposed with the 1AD3 complex aligning structurally the proteins to locate the coenzyme inside each ALDH model. This initial geometry was then optimized minimizing the energy during 500 steepest descent steps with UCSF Chimera 1.11 [55] to obtain final structures of ALDH-NAD complexes. The initial geometry of crocetin dialdehyde was taken from PubChem database (PubChem Compound Identifier (CID) 11109238) without optimizing it because the docking calculations performed (see below) treat ligands as flexible molecules. The docking solution gives thus ligand geometries suited to every protein environment.

### 3.6. Docking

The coordinates of phosphorus PA atom of NAD coenzyme (approximately the center of the coenzyme) in each ALDH–NAD model complex were selected to define the center of a 25 Å cubic grid box for geometry search in the docking calculations intended to dock crocetin dialdehyde. The best solution i.e., that having the lowest protein–ligand affinity free energy, was selected as the final model structure of each ALDH–NAD–crocetin dialdehyde complex. Protein, coenzyme and ligand structures were prepared for docking (generation of PDBQT files) with UCSF Chimera 1.11. Docking calculations were performed with AutoDock Vina [56]. Analysis of structures and visualization were carried out with UCSF Chimera 1.11 and PyMOL 1.8.4 [57].

## 4. Conclusions

Taking together all the data, the four ALDHs from *C. sativus* can use the crocetin dialdehyde to produce crocetin, with ALDH54788 and ALDH11637 showing more affinity to the substrate in comparison to ALDH20158 and ALDH3898. ALDH11637 and ALDH20158 were found to be more tissue-specific since the transcript were detected only in the stigma while the ALDH54788 and ALDH3898 were detected in more tissues. Phylogenetic analysis and binding affinities calculations pointed out that members of family 2 C4 from different species accumulating crocetin have high affinity toward crocetin dialdehyde and results revealed that the ALDH Q70SZ7 from saffron appears to be the specific one; however, it seems that any ALDH can act over crocetin dialdehyde and produce crocetin no matter its location, expression pattern, or the family in which is integrated.

## Figures and Tables

**Table 1:** Transcript levels of the ALDHs in different saffron organs detected by qPCR analysis.

Gene	Red Stigmas	Leaf	Root	Cormlet
*ALDH20158*	69.7 ± 1.2	nd	nd	nd
*ALDH3898*	29.1 ± 0.6	65.3 ± 0.9	73.7 ± 0.5	nd
*ALDH54788*	132.5 ± 2.1	99.5 ± 1.4	150.5 ± 1.5	nd
*ALDH11367*	101.4 ± 1.1	nd	nd	nd

nd: not detected.

**Table 2:** Structural comparison between ALDH models and template proteins used for homology modelling.

Aldehyde Dehydrogenase	Template (Protein Data Bank:PDB id)	Seq. Identity (%)	Residues in the Superposition/Total Residues	Root-Mean-Square Deviation (RMSD) Backbone Atoms (Å)
ALDH54788	4QGK	43.61	452/459	0.295
ALDH3898	4ZZ7	46.94	480/488	0.272
ALDH20158	5ABM	55.79	483/485	0.344
ALDH11367	5ABM	55.09	481/481	0.212

**Table 3:** Protein–ligand affinity free energies for the best geometries obtained in AutoDock Vina calculations for docking of crocetin dialdehyde to ALDH–NAD complexes.

ALDH	ΔG^0^ (kcal/mol)	K_d_ (μM)
ALDH54788	−5.8	56.0
ALDH3898	−5.3	130.0
ALDH20158	−5.5	92.9
ALDH11367	−5.8	56.0

**Table 4:** Protein–ligand affinity free energies for the best geometries obtained in AutoDock Vina calculations for docking of crocetin dialdehyde to ALDH–NAD complexes.

ALDH	ΔG^0^ (kcal/mol)	K_d_ (μM)
CAD70189.1 (*Bixa Orellana*)	−7.3	4.2
c74954_g1_i1 (*Crocus sieberi*)	−8.0	1.3
KY631926 (*Gardenia jasminoides*)	−9.0	0.2
MH182707 (*Buddleja davidii*)	−8.5	0.5
Q70SZ7 (*Crocus sativus*)	−8.2	0.9

## Supplementary Materials

Supplementary materials can be found at http://www.mdpi.com/1422-0067/19/5/1409/s1.
