# Supplementary material for: Expression and Interaction Analysis among Saffron ALDHs and Crocetin Dialdehyde
Source: Int J Mol Sci. 2018 May 9;19(5):1409. doi: 10.3390/ijms19051409 (PMC5983644; doi:10.3390/ijms19051409)
Supplement: Supplementary file 1 [file ijms-19-01409-s001.pdf]

Supplementary materials

Expression and Interaction Analysis among Saffron ALDHs and Crocetin Dialdehyde

Lourdes Gómez-Gómez [1], Luis F. Pacios [2], Araceli Diaz-Perales [2], María Garrido-Arandia [2], Javier Argandoña [1], Ángela Rubio-Moraga [1] and Oussama Ahrazem [1,3,*]

[1] Instituto Botánico, Departamento de Ciencia y Tecnología Agroforestal y Genética, Facultad de Farmacia, Universidad de Castilla-La Mancha, Campus Universitario s/n, 02071 Albacete, Spain; Marialourdes.gomez@uclm.es (L.G.-G.); javier.argandona@uclm.es (J.A.); angela.rubio@uclm.es (A.R.-M.)

[2] Centro de Biotecnología y Genómica de Plantas (CBGP, UPM-INIA), Universidad Politécnica de Madrid (UPM)—Instituto Nacional de Investigación y Tecnología Agraria y Alimentaria (INIA), Campus de Montegancedo-UPM, 28223 Pozuelo de Alarcón, Madrid, Spain; luis.fpacios@upm.es (L.F.P.); araceli.diaz@upm.es (A.D.-P.); maria.garrido@upm.es (M.G.-A.)

[3] Facultad de Ciencias Ambientales y Bioquímica Toledo, Campus Tecnológico de la Fábrica de Armas, Avda. Carlos III, s/n, E-45071 Toledo, Spain

* Correspondence: oussama.ahrazem@uclm.es; Tel.: +34-967-599-200

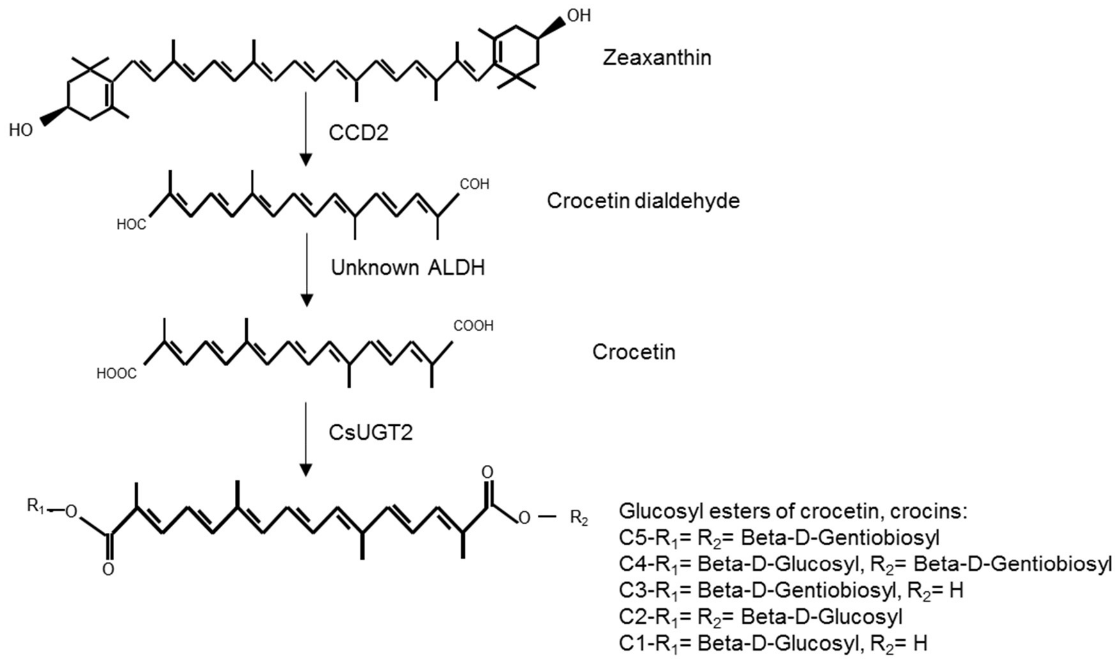

Figure S1. Crocins biosynthesis pathway from zeaxanthin. CCD2: Carotenoid cleavage dioxygenase family 2, ALDH: Aldehyde dehydrogenase and CsUGT2: UDPG-Glucosyltransferase 2

Primers used for qPCR

CsADH-3898_F: ACGTCAGGTGTGTCTGCAAG

CsADH-3898_R: TGTGGGCATAGCCAATGATA

CsADH-11367_F: CTGATGAGGGCATTGAAGGT

CsADH-11367_R: TTTCGTCGACGTCTTCTCCT

CsADH-20158_F: CCGGTCCAAATTAACCACAC

CsADH-20158_R: GCTCGAAACAGTATGCGTGA

CsADH-54788_F: CAGGGAGAGCTTCAACAAGG

CsADH-54788_R: TTTGAGCTGGCACCTTTTCT

Primers used in pThio-Cloning

CsADH-3898_F CGCCCTTGGCGAATTATGCTTCAATCTCCTCTCCGACG

CsADH-3898_R TACCCTCGAGGAATTTTACGACTTCTGGGATGTGGGC

CsADH-11367_F CGCCCTTGGCGAATTATGGCTGCTGCGAAGAGG

CsADH-11367_R TACCCTCGAGGAATTAGCATATCAATTTTCTCTCTTGCCTGC

CsADH-20158_F CGCCCTTGGCGAATTATGGCGGCTGCCAAGAAG

CsADH-20158_R TACCCTCGAGGAATTCTACAACCATGCTGGATTCTTGAGG

CsADH-54788_F CGCCCTTGGCGAATTATGGCCTTTGATGGAGAGAAAGCT

CsADH-54788_R TACCCTCGAGGAATTTCAAGATTTGGAGAAACCCAATAGAGCC

Sequences used to generate the phylogenetic tree.

>CAD70189.1 (Bixa Orellana)

MESTGRAPLGNGRIGNPGGLVVPEIKFTKLFINGRFVDNVSGQTFDTRDPRTGDVLATIAEADKDDLDLAVK
NAREAFDHGPWPRMSGYERARIMTKFADLVEAFIEELAALDTADAGKTLSMGKAVDIPAAVQIMRYYAGA
ADKLHGQPLKMNQDTQGYTLKDPIGVVGHIIPWNYPTMMFFLKNAPALAAGCTMVVKPAEQTPLSALFY
AHLARMAGVPDGVVNVVPGFGPTAGAAVSSHMDVDSVSFTGSQEVGRAIMRAAAQSNLKNVSLELGGK
SPVIIFDDADVDMAVSLSQLACFTNKGEICVATSRVYVQEGIYDALVKKIVEAAREWRFDTKANMGPQVDK
KQFERVLKYIDLGKREGATLLTGGKTNGDKGYYIQPTIFLDVREDMNIAQDELFGPVMALMKFKTVDEAIQK
ANCTKYGLAAGIVTKELNLANTVSRSIRAGAVWVNCYFGFDPDAPFGGYKMSGFGKDRGVLAIDQYLKAKT
VNTAIPNSPWY

> c74954_g1_i1 (Crocus sieberi)

MANNCNGNSGEELKIPEIKYTKLFINGQFTDSVSGKTFETIDPRNGEVIAKLAEGDKADVDLAVKAAREAFD
HGKWPRMSGYERGRIMMKYADLIEEHLEEVANLDCLDGGKVFTQVKLRDIPGAAQLLRYNAGAADKIHGE
TLKLSGEFQGYTLKEPIGVVGHIIPWNYPSTMFFLKVSPALAAGCTMIVKPAEQTPLSALFYAQLAKKAGIPD
GVINVVTGFGTTAGSALSHHMDVDKISFTGSTEVGRLVMEAAARSNLKHVSLELGGKSPVIIFDDADVDMA
VGLAQMATFYNKGEICVAGSRIYVQEGIYDEFVKKIAEKTKSWVVGDPFDPNVQQGPQVDKVQFEKVLRYI
EYGKAEGATLLTGGKACGEKGYYIEPTIFTDVKEDMKICQEEIFGPVMSLMKFKTTEEAIEKANNTKYGLAAG
ILTNDLNTANRVSRSIRAGTIWINCYFSFDRDCPFGGFKMSGFGKDMGLQAIEKYLQEKCVVTPIYGSPWL

>KY631926 (Gardenia jasminoides)

MAVQSNGNGSSDSHVKVPQIKFTKLFINGEFVDSVSGKTFETIDPRNGEVIAEIAEGGKEDVDLAVKAAREA
FDHGPWPRLPGSERRKIMMKFADLIDENTQELATLDAIDAGKLFYLCKIMDIPGAAETIRYYAGAADKIHGE
TLKMSTALQGYTLHEPIGVVGHIIPWNFPSQMFAMKVGPALAAGCTMVVKPAEQTPLSALYYAHLAKLAGI
PDGVLNVVTGFGQTAGAAISSHMDIDMVSFTGSTEVGRLVMQAAATSNLKPVCLELGGKSPILIFDDADVD
KVTELALQGTLFNKGEICVAGSRIFVQEGIYDKFLIKLKQKVKNWVVGDPFDPTSHQGPQVDKKQYDRILSYI
EHGKKEGATLFHGGKPCDGKGYYIEPTIFTDVTDEMTIAKEEIFGPVMSVFKFKTVEEAIKRANATKYGLAAG
VMTNNINIANTVARSIRAGAIWINCYFAFDRDSPYGGYKMSGFGRDMGMDGLKKYLAVKAVATPIYNSPW
L

>KY631927(Gardenia jasminoides)

MAAKRISSLLLSRSLTPSLASRLTRGKSACAGAISRYSTAAAALEEPIKPPVSVEYTQLLINGQFVDAASGKTFP
TLDPRTGEVIAHVAEGDAEDINRAVAAARKAFDEGPWPKMTAYERQRILLRFADLVEKHNDEIAALETWDN
GKPYEQAAKIEVPMFVRLIRYYAGWADKIHGMTVPADGPHHVQTLHEPIGVAGQIIPWNFPLLMYAWKV
GPALACGNTIVLKTAEQTPLSALYVTKLLHEAGLPSGALNVVSGFGPTAGAALCSHMDVDKLAFTGSTETGK
VILELSAKSNLKPVTLELGGKSPFIVCEDADVDNAVELAHFALFFNQGQCCCAGSRTYVHERVYDEFVEKATA
RALKRSVGDPFRQEIEQGPQVDSEQFQKILRYIRAGVDGGATLETGGDRLGEKGYYIKPTVFSNVQDDMLIA
TDEIFGPVQSILKYRDLDEVIRRANSSRYGLAAGVFTQSLDTANTVMRALRVGTVWINCFDTFDAAIPFGGY
KMSGQGREKGEYSLKNYLQVKAVVTPLKNPAWL

>KY631928 (Gardenia jasminoides)

MSLFEEITIQQVGITYKQPLGLFINNEYTASSDGGKIQTINPATEEYITSFYAGTEEDADRAVGAAREAFENHW
RKTSPKERGDLLYKLANIVEREKVLLAALETLDSGKPYHSNSLGDINEVIKVTRYFAGAADKFNQGNISSSNHD
KYGYSLKVPYGVVAHVIPWNYPLLMACWKLQGCLAAGNCIVVKPAENTSLSLLYFAQLVKEAGFPPGVFNV
VPGYGPVAGSRLALHPDVDKIGFTGSTTVGKKIMSMAGQSNLKDVTLECGGKTAAIVLGDAILEDAIDWLTL
GIFFNSGQNCTANSRIYVQESIFDEFLSKFKENTFKNWKFGNNYDPFDPECTMGPLISKVQYDRVSGYLSHG
KETEKLEAIELGVVKNGIGFFVPPTIFINVPQDSKLCKEEIFGPVAVISTFKNYDEAVALANDSDYGLASCIFTEN
VRKANQFVRDARSGTVYVNCSNEDDISLPFGGFKMSGIGRELGYAGMESYLQLKSVHMNIGNPSFL

>XP_957628 (Neurospora crassa OR74A)

MAASKVEIAPFEVTPLDAIPAVCSTARATFASHKTKNLQWRLVQLRKLYWALDDFKASLMAALQQDLRKG
GYESDFTEVDWVKNDCLHMINNLETFAKTEKLKDLPVTYSMMNFRVKKEPLGTVLIIGPYNFPIQLVLAPLV
GAIGAGCTAVIKPSELTPACAMAMKEMIESRLDRDAFAVVNGGVPETNALMEEKWDKIMFTGSAQVGSII
ARKAAETLTPVCLELGGRNPAFVTKKANLALAARRLMWGKVLNAGQVCMSHNYVLVDKDVADTFIEFLKIA
YKDMFPNGAKASPDLSRIVNARHFNRIKKMLDETKGKIVMGGEMDESELYIEPTAVLVDSLDDPMMQEES
FGPIFSIYPVDTLDQALSIANNVHRTPLALMAFGDKSETNRILDEMTSGGACINDSYFHGAVHTVPFGGVGD
SGWGAYRGKASFDNFTHFRTVSETPTWMDRFLRVRYMPYDWSELRLLQRWTNKKPNFDRQGTVAKGSE
YWMWYFLGLGTKGGVKGALMRWLVVVAGYYLSAYMKARRA

>NP_000680 (Pan troglodytes)

MSSSGTPDLPVLLTDLKIQYTKIFINNEWHDSVSGKKFPVFNPATEEELCQVEEGDKEDVDKAVKAARQAFQ
IGSPWRTMDASERGRLLYKLADLIERDRLLLATMESMNGGKLYSNAYLNDLAGCIKTLRYCAGWADKIQGR
TIPIDGNFFTYTRHEPIGVCGQIIPWNFPLVMLIWKIGPALSCGNTVVVKPAEQTPLTALHVASLIKEAGFPPG
VVNIVPGYGPTAGAAISSHMDIDKVAFTGSTEVGKLIKEAAGKSNLKRVTLELGGKSPCIVLADADLDNAVEF
AHHGVFYHQGQCCIAASRIFVEESIYDEFVRRSVERAKKYILGNPLTPGVTQGPQIDKEQYDKILDLIESGKKE
GAKLECGGGPWGNKGYFVQPTVFSNVTDEMRIAKEEIFGPVQQIMKFKSLDDVIKRANNTFYGLSAGVFTK
DIDKAITISSALQAGTVWVNCYGVVSAQCPFGGFKMSGNGRELGEYGFHEYTEVKTVTVKISQKNS

>NP_000684 (Homo sapiens)

MATANGAVENGQPDRKPPALPRPIRNLEVKFTKIFINNEWHESKSGKKFATCNPSTREQICEVEEGDKPDV
DKAVEAAQVAFQRGSPWRRLDALSRGRLLHQLADLVERDRATLAALETMDTGKPFLHAFFIDLEGCIRTLRY
FAGWADKIQGKTIPTDDNVVCFTRHEPIGVCGAITPWNFPLLMLVWKLAPALCCGNTMVLKPAEQTPLTA
LYLGSLIKEAGFPPGVVNIVPGFGPTVGAAISSHPQINKIAFTGSTEVGKLVKEAASRSNLKRVTLELGGKNPCI
VCADADLDLAVECAHQGVFFNQGQCCTAASRVFVEEQVYSEFVRRSVEYAKKRPVGDPFDVKTEQGPQID
QKQFDKILELIESGKKEGAKLECGGSAMEDKGLFIKPTVFSEVTDNMRIAKEEIFGPVQPILKFKSIEEVIKRAN
STDYGLTAAVFTKNLDKALKLASALESGTVWINCYNALYAQAPFGGFKMSGNGRELGEYALAEYTEVKTVTI
KLGDKNP

>Np033048 (Mus musculus)

MTSSEIAMPGEVKADPAALMASLQLLPSPTPNLEIKYTKIFINNEWQNSESGRVFPVCNPATGEQVCEVQE
ADKVDIDKAVQAARLAFSLGSVWRRMDASERGRLLDKLADLVERDRATLATMESLNGGKPFLQAFYIDLQG
VIKTLRYYAGWADKIHGMTIPVDGDYFTFTRHEPIGVCGQIIPWNFPLLMFTWKIAPALCCGNTVVIKPAEQ
TPLSALYMGALIKEAGFPPGVVNILPGYGPTAGAAIASHIGIDKIAFTGSTEVGKLIQEAAGRSNLKRVTLELG
GKSPNIIFADADLDYAVEQAHQGVFFNQGQCCTAGSRIFVEESIYEEFVKRSVERAKRRIVGSPFDPTTEQGP
QIDKKQYNKVLELIQSGVAEGAKLECGGKGLGRKGFFIEPTVFSNVTDDMRIAKEEIFGPVQEILRFKTMDEVI

ERANNSDFGLVAAVFTNDINKALMVSSAMQAGTVWINCYNALNAQSPFGGFKMSGNGREMGEFGLREY
SEVKTVTVKIPQKNS

>NP038495 (Mus musculus)

MSSPAQPAVPAPLADLKIQHTKIFINNEWHNSVSGKKFPVLNPATEEVICHVEEGDKADVDKAVKAARQAF
QIGSPWRTMDASERGRLLNKLADLMERDRLLLATMEALNGGKVFANAYLSDLGGCIKALKYCAGWADKIH
GQTIPSDGDIFTYTRREPIGVCGQIIPWNFPMLMFIWKIGPALSCGNTVVVKPAEQTPLTALHLASLIKEAGF
PPGVVNIVPGYGPTAGAAISSHMDVDKVAFTGSTQVGKLIKEAAGKSNLKRVTLELGGKSPCIVFADADLDI
AVEFAHHGVFYHQGQCCVAASRIFVEESVYDEFVKRSVERAKKYVLGNPLTPGINQGPQIDKEQHDKILDLIE
SGKKEGAKLECGGGRWGNKGFFVQPTVFSNVTDEMRIAKEEIFGPVQQIMKFKSVDDVIKRANNTTYGLA
AGLFTKDLDKAITVSSALQAGVVWVNCYMMLSAQCPFGGFKMSGNGRELGEHGLYEYTELKTVAMKISQK
NS

>fr850689 (Fusarium fujikuroi)

MAAAGFDYGCQQSSYTIAPLEHTPLDEIAAKVDLVRKTFRSGRTKDMEFRMKQIRKLYWAIVDNTELMQD
ALIKDLRKCKYEAVLAEIDWCKQECIDMVNNMEKWLRDEPVPNVPLQFRAMKHRTRFEPLGVVLNIGSFNF
PFQLNLPVVIGAIACGNCVVLKASESSPNCAMVLKKIFDESLDPECFTYVNGALPETQLLLEQKFDKICFTGGK
AVGKIIAQKAAETLTPVLLELGGLNPAFVTKHANLKLAARRLLWQKSLNAGQVCMSHNYILVERGVLSQFLG
ELNNQMRTFFPQGAKNSPDLCRIVNAGHFNRLKKMLDGTNGKIVLGGSMDESTLFMEPTAVLVDDINDS
MMTQEAFGPIFAMMAVDSLDQAIDIANTVDPTPLSLSAFGSKAENNKILDNVTSGGATCNDAFFHSQIPQS
PLGGVGQSGMGNYHGIYSIRTFSHQRTIAEVPYWADFLFRVRYMPYQWPVMNRMKAVADSKPNFDRNG
NKTKGITYFLALVLGLGSKKSKGALLRWAVLVVAAAILEAKKGVLSQLLTR

>WP_012263921 (Microcystis aeruginosa)

MLSNSEKLAKLRQYFASGATRSYQFRRQQLEKLKQAIIKYEAEIYRALYSDLKKSPEDCWVTENGFLLVEINTA
LKNLRSWMQPKTVKTNLLNFPSSSQIIREPLGVVLIIGAWNYPLQLLLVPLVGAIAAGNCAVLKPSEFASATEK
LVVKIIEEIFPEEYVLIVPGDGAEVIPNMLDSFTFDHIFFTGSTRVGKIIYQLAAAKLIPVTLELGGKSPCVIEADA
NISVATRRIAVTKFSNCGQMCIAADYVLVHQSQQENVIRELVNTIVNFFGKDARLSADYGKIINEKQFDRLID
FLQDGEIVFGGKTDRENLYIQPTLLTNVSLDAPIMQGEIFGPILPIIAFSTFEEALAIIAKNPNPLAFYLFTTKAKT
EKKWLESVQFGGGCINNNSFHFTNPSLPFGGRGNSGIGSYHGRFSFENFSHQKAIMKTPLWFNPVLKYPPF
KGKLNLFKLLVR

>XP_006340688.1 (Solanum tuberosum)

MNGVEEDVLGELRTTFRSGRTRSVAWRKAQLQAILKLLDENEEEIFEALKQDLGKHPVESYRDEVGVVRKSA
TNALRCVEKWMAPQKAPIPLVLFPARGAVVSEPLGVVLLFVSWNFPISLTLDPAIGAISAGNTIVLKPSELAPK
CSSVLANTIPRYLDPEAIKVVEGGQDVSEQLLQLKWDKIFFTGSPRVGRLIMSAAAKHLTPVTLELGGKCPTIL
DTLSSSYDLQVAVKRIAGGKWGPCNGQACIGIDYVLVETQFAPVLIELLEKIIKTFYGENLKTLGNLARIVNKH
HFDRVHNLLKDPKVAASVVYGGSVDEENMVIEPTILLNPPLDADIMTEEIFGPLLPIITLNNIEESIQFINSRPKP
LAIYAFTKNDSLKEKILQETSSGSLTFNDAMIQFICDTLPFGGVGQSGYGRYHGKFSFDTFSHEKAVLHRSLLIE
LESRYPPWNNFKLEFVRLAYDYDYLGLILLLLGLRGIFRTNRRQ

>BAV82469.1 (Cocos nucifera)

MSGAIPRRQLFIDGEWRDPVRQKRIPVINPATEEPIGDIPAATAEDVELAVAAARRALTRNRGKDWARTSG
AARAKYLRAIAAKITERKSELAKLETLDCGKPLDEAAWDMDDVAGCFEYYADLADALDGKQRAPVSLPMQT
FKSYVLKEPIGVVGLITPWNYPLLMASWKVAPALAAGCTAVLKPSELASVTCLELADVCKEVGLPPGVLNIVT
GFGPEAGAPLASHPHVDKIAFTGSTETGRRIMTAAAQTIKPVSLELGGKSPIIVFEDVDIEKAVEWTLFGCFW
TNGQICSATSRLLVHESISKEFMERLVAWAKNIKISNPLEEGCRLGPVVGEGQYEKIKKFISTAKSEGATILCGG
GRPQHLEKGFFIEPTIIKDVDASMQIWREEVFGPVLCIKTFGTEEEAVELANDTHFGLAGAVISKDPERCNRA
SEEIQAGIVWINCSQPCFCQAPWGGNKRSGFGRELGEWGLDNYLSVKQVTEYISDEPWAWYPPPSKL

>OAY76151.1 (Ananas comosus)

MASPPLEVPRRGLFIDGEWREPLLKKRIPIINPASEAVIGDVPAATAEDVDLAVEAARRALSRNRGRDWARA
SGAARAKHLRAIAAKITERKSQLAKLEAIDCGKPLDEAAWDIDDVAGCFEYYADLAEALDKKQKAPISLPMET
FKSYVLKEAIGVVALITPWNYPLLMATWKVAPALAAGCTAILKPSELASVTCLELADVCKEVGLPPGVLNIVT
GLGPDAGAPLAAHPHVDKIAFTGSTETGRRIMTAAAQLVKPVSLELGGKSPLIVFDDVDIDKAVEWALFGCF
WTNGQICSATSRLLIHEKIAKQFLERLVAWAKNIKVSDPLEEGCRLGPVVNEGQYEKIKKLISTAKNEGATILS
GGVRPKHLEKGFFIEPTIITDVNTSMQIWREEVFGPVLCVKEFSTEEEAIELANDTQYGLGGGVISNDLERCQ
RLAEEIQAGIVWINCSQPCFCQAPWGGNKRSGFGRELGEWGLENYLSVKQVTQYVSDDPWGWYVSPSKL

>XP_003544699.1 (Glycine max)

MASNTKSKALFDSEEALMMVKELRVTFDCGKTRSYEWRISQLKAIIKLTEENEQQIYQALHSDLSKCETEAFV
QEIAMLKNSCRIALKELKHWMTPEKVKTSIATFPSSAEIVSEPLGVVLVISAWNYPFLLSLDPVIGAIAAGNAV
VLKPSEIAPATSSLLAKLLGDYLDNSCIKVVEGAVDETSALLQQKWDKIFYTGNGRVARIVMAAASKHLTPVV
LELGGKSPVVVDSNINLKVATRRIIAGKWGSNNGQACISPDYIITTKDYAPKLVDALKTELEKFYGKNPLESKD
LSRIVNSNHFNRLTKLLDDDKVSGKIVYGGEKDESKLKISPTVLLDVPRDSLIMNEEIFGPLLPILTVDKIEESFD
VINSGSKPLAAYIFTNTKKLKEQFVMTISAGGLVVNDTTLHLAVHTLPFGGVGESGVGAYHGKFTFEAFSHKK
AVLYRRFIGDAPVRYPPYTNTKMRLLKALIGGGILGIIRALFGW

>ATW64820.1 (Zea mays)

MSRLLSRQHLAAVRRSAPFACVSRWLHTPSFATVSPQEVSGSSPAEVQNFVQGSWTASANWNWIVDPLN
GDKFIKVAEVQGTEIKSFMESLSKCPKHGLHNPLKAPERYLMYGDISAKAAHMLGQPTVLDFFAKLIQRVSP
KSYQQALAEVQVSQKFLENFCGDQVRFLARSFAVPGNHLGQRSNGYRWPYGPVAIITPFNFPLEIPLLQLM
GALYMGNKPVLKVDSKVSIVMEQMIRLLHDCGLPAEDMDFINSDGAVMNKLLLEANPKMTLFTGSSRVAE
KLAADLKGRVKLEDAGFDWKILGPDVQEVDYVAWVCDQDAYACSGQKCSAQSVLFMHKNWSSSGLLEK
MKKLSERRKLEDLTIGPVLTVTTEAMIEHMNNLLKIRGSKVLFGGEPLANHSIPKIYGAMKPTAVFVPLEEILK
SGNFELVTKEIFGPFQVVTEYSEDQLELVLEACERMNAHLTAAIVSNDPLFLQDVLGRSVNGTTYAGIRARTT
GAPQNHWFGPAGDPRGAGIGTPEAIKLVWSCHREIIYDVGPVPESWALPSAT

>XP_002441445.1 (Sorghum bicolor)

MSRLLSRRQIDAVRRSAPLACVSRWLHTPSFATVSPHEVSGSSPAEVQNFVQGSWTASANWNWIVDPLN
GDQFIKVAEVQGTEIKPFVESLSKCPKHGLHNPLKAPERYLMYGDISAKAAHMLGQPAVSDFFAKLIQRVSP
KSYQQALAEVQVSQKFLENFCGDQVRFLARSFAVPGNHLGQRSNGYRWPYGPVAIITPFNFPLEIPLLQVM
GALYMGNKPVLKVDSKVSIVMEQMIRLLHDCGLPAEDMDFINSDGVTMNKLLLEANPKMTLFTGSSRVAE
KLAADLKGRVKLEDAGFDWKILGPDVQEVDYVAWVCDQDAYACSGQKCSAQSVLFMHKNWSSSGLLEK
MKKLSERRKLEDLTIGPVLTVTTEAMIEHMNNLLKIQGSKVLFGGEPLANHSIPKIYGAMKPTAVFVPLEEILK

SGNFELVTKEIFGPFQVVTEYSEDQLELVLEACERMNAHLTAAVVSNDPLFLQDVLGRSVNGTTYAGIRART
TGAPQNHWFGPAGDPRGAGIGTPEAIKLVWSCHREIIYDVGPVPKSWALPSAT

>PKA47055.1 (Apostasia shenzhenica)

MGFAKEHRFLSEIGLGPRNPGCYVNGIWRGSGPVVASLNPADNQVIAEVVEASVEDYEEGMKACSDASQI
WMQLPAPKRGDIVRQIGDALRAKLLHLGRLVSLEMGKILPEGIGEVQEIIDMCDFAVGLSRQLNGSIIPSERP
NHLMMEVWNPLGIVGVITSFNFPCAVLGWNACIALVCGSCVVWKGAPTTPLITIAMTEIIAGVLENNNLPG
AIFTSFCGGADIGQLIARDSRISLVSFTGSSKVGLMVQKTVNERFGKCLLELSGNSAIIVMDDADVQLAVRSVL
FAAVGTAGQRCTTCRRLLLHESIYQSVLDQLVGVYKQVKIGDPLEKGTLLGPLHTAASKENFVHGIEVIKSQG
GKILVGGSVIKSEGNFVQPTIVEISPDAPVVKEELFGPVLYVMKFQTLKEAIEINNSVPQGLSSSIFTHRPEIIFK
WIGPLGSDCGIVNVNIPTNGAEIGGAFGGEKATGGGREAGSDSWKQYMRRSTCTINYGSELPLAQGINFG

>Q70SZ7 (Crocus sativus)

MVSTGCSGNGAKGTNGGIVVPEIKFTKLFINGEFVDSVSGSTFETRDPRNGDVIANIAEGDKEDVDLAVKAA
REAFDHGKWPRMSGYERGRIMMKFADLIEAFIEELAALDTLDAGKLLSMGKAVDIPAAVHIIRYYAGAADKI
HGYTLKLSSELQGYTLKEPIGVVGVIIPWNFPTTMFFLKVSPALAAGCTMVVKPAEQTPLSALYYAHLAKLAG
VPDGVINVVPGFGPTAGAALSSHMDVDSVAFTGSAEIGRAIMESAAKSNLKNVSPELGGKSPMIVFDDADV
DMAVSLNSLAVFFNKGEVCVAGSRVYVQEGIYDEFVKRAVEAARSWKVGDPFDQSRNMGPQVDKDQFES
VLKYIEHGKSEGATLLTGGKPAADKGYYIEPTIFVDVTEDMKIAQEEIFGPVMSLMKFKTVEEGIDCANNTKY
GLAAGILSQDLDLINTVSRSIKAGIIWVNCYFGFDLDCPYGGYKMSGNCRESGMDALDNYLQTKSVVMPLH
NSPWL

>XP_009411491.1 (Musa acuminata subsp. Malaccensis)

MESVGVEGVANEVRTDYESGKTRSLAWRRSQLKALQRLLYEEEEGMFTALKQDLGKHQAEAYRDEVGILIK
SVNYALENLKKWMTPCRVPVPLLAFPTRGELVPEPLGVVLIFSSWNFPIGLSLEPLIGAIAAGNAIVLKPSELAP
ASSNFLAKSVPKYLDSMSVKVVRGGPDVGQKLLEQKWDKIFFTGSSRVARIVMAAAAKHLTPVAVELGGKS
PAIFDSLSSARDRKVAVERIVGAKWVPCSGQVCIGVDYVLVEEQFAPILIDQLKATLEKFYPRFDCLSRIINEQH
FQRLSNLIKDPSVAATIIHGGSLDSETLFIEPTILLDPPLDAEIMNEEIFGPLLPIITLKKIEDSIEFIRARPKPLVIYAF
TKDEKLRSRITAETSSGSLTFNDTMIQFACDELPFGGVGGSGFGQYHGKFSFDMFSHTKAVLRRSFLLEFTFR
YPPWDAWKLPFMRSVYHFDYVTLLLRLLGLKRG

>XP_008598050.1 (Beauveria bassiana ARSEF 2860)

MASSGAIPAYEPTSLDNITASIDTIRKTFRTNKTKDVDFRIRQIRKLYWGVADNAALIEEALMKDLSKCKFEAQ
LSEIDWIKNEAVYVSNSVKKWAKDKAVDDIPFQYWALGPKMRSEPYGTILIIGAFNYPFQLNLAPVIGAIAAG
NTVLLKPSELSPHSAMVLKKIFDEYLDPDCYVCVNGNVDETKHIMEHKFDKIVFTGGKGVGTIIAKKAAETLT
PYILELGGQNPSFVTRHADVKLAAKRLMWQKTFNAGQVCLSHNYVLVERPVLNEFVAEINKLHAKFMPHG
ADKSPDWSRIVNMNHFNRLKRMLDNTKGKIVMGGNMWEDKLIIEPTAVLVDSIDDSMMVEESFGPIWSI
LPFDTLDEAIDIANKVDPTPLSLTTFGSDAENEKILLNVTSGGATINDGFWHAAMNQTPIGGVGSSGQGNY
HGAFSFKAFSHERIICKVPKWADLVLKLRYMPYDLKELARTQALTSLTPNFDRDGNPKRGLFYWIGLIFTLGG
RGSKKSSILRWAILMPVVAYLVELRRAKL

>NP_442494 (Synechocystis sp. PCC 6803)

MNTAKTVVAEQRDFFRQGKTKSVQDRLTALAKLKTQIQAQEEEIIKALKQDFGKPTFESYVNEILGVIREINYY
QKHLQQWSKPQRVGTNLMVFPASAQLRPEPLGVVLIISPWNYPFYLCLMPLIGAIAAGNCVVVKPSEYTPAI
SGVITRLIQNVFSPAWATVVEGDETISQQLLQEKFDHIFFTGSPRVGRLIMAAAAEQLTPVTLELGGKSPCVV
DREINLQETAKRIMWGKLVNAGQTCVAPDYLLVEQSCLEQLLPALQQAIQMLFGENPAHSPDYTRIVNQQ
QWSRLVSLLSHGKVITRGDHNEGDRYIAPTLIIDPDLNSPLMQEEIFGPILPILTYQSLSEAIDFINIKPKPLALYF
FSNNRQKQEEILQSTSSGSVCLNDILLHLTVTDLPFGGVGESGMGRYHGKATFDTLSNYKSILRRPFWGETNL
RYSPYGKKMNLIKKLFS

> XP_020265822.1 (Asparagus officinalis)

MVAPLASWPWSNLGSCKYLLYGPLVAKVLYSRYNVDRGISVESSSWCLHLLALSLLRGFIHQAWFSFSSMLF
LTRRRRIIKDGVDFEQIDKEWDWDNFLILQVFMGAALFYMFPSLINLPIWDFRGLIMAALLHVSVSEPLFYCA
HRLLHRGPLFEKYHSTHHSSKVLQSFTAGFGTPLEHLVMSIVMGAPVVGANLMGFGSMGLVYGYILFFDFM
RCLGHSNVEVFPHRIFHAFPFLRYIVYTPTYHSLHHTEKNSNFCLFMPLFDLLGGTLNDRSWELQKEISSAKD
DQVPAFVFLAHVVDVAFSLHSPFILRSFSSSPFSVRLWILLLWPGAFIIMLFMWAFSKTFLLSFYNIRGRLHQT
WLVPRYGFQYFLPFAKDGINDQIELAILRADKMGIKVLSLAALNKNEALNGGGTLFVNKHPNLKVRVVHGN
TLTAAVILNEIPPNVGEVFLTGATSKLGRAIALYLCRKKIRVMMLTLSTERFLKIQKEAPVEFQQYLVQVTKYQ
AAQNCKTWIVGKWLTPREQRWAPRGTHFHQFVVPPIISFRRDCTYGKLAAMRLPKDVQGLGMCEYKLDR
GVVHACHAGGVVHSLEGWTHHEVGAIDVDRIDIVWRAALKHGLKPV

>MH182707 (Buddleja davidii)

MSSTGQTNGASILLDEYSHNIKIPKIKFTKLFINGHFLHSISGNTFETIDPRTEEVIANIAEGDKDDVDLAVNAA
RQAFDHGPWPRLPGSERRRIMLKFADLIDENVEELAALDAIDGGKLYKMAKLADIPGAAETIRYFAGAADKI
HGATLKMSRALQGYTLREPIGVVGHIIPWNFPSQMFVMKVGPALAAGCTMVVKPAEQTPLSALYYAHLAK
LAGIPDGVVNVVTGYGHAAGAAISSHMDIDMVSFTGSTEVGRLVMQAAAQSNLKPVSLELGGKSPFIVFD
DVDVDKVADLALQATLYNKGEICVAGTRVFVQEGIYDKFLTKLVEKAKAWVVGDPFDPNVHQGPQVNKK
QYERILSFIELGKKEGATLLTGGKPCDRKKGYYIEPTIFTDVDDDMTIAKEEIFGPVMSVMKFKTVEEVIKRAN
ASKYGLATGIMTNNLNIANTVSRSIRAGVIWINCYFAFDRDLPYGGYKMSGFGRDLGMESLSKYLQVKSIAT
PIYDSPWL

>XP_020263644.1 (Asparagus officinalis)

MAAAGLEAVLNEVRDTFEGGKTKSLSWRKSQLNALLRLLVETEDEIFRVLKEDLGKHRVEAFRDEIGFLLKSV
NYHLDNIKKWMAPKKVDIPIAAFPSTCELVAEPLGVVLLISSWNFPIGISLEPLIGAISSGNAVVLKPSELAPSSS
AFLANTIPKYLDPKAVKVIEGGPRIGERLLEHKWDKIFFTGSTRIGRMVMAAAAKHLTPVALELGGKCPAIVD
YLSSSRDKKVAVERVVAGKWGSCNGQACVAIDYLLVEEKFAPILVEMIKGTLKRFYPKPDDTAKIVNEHHFQ
RLSNLLKNPSVTSSIVHGGSVDYKNQTIEPTVVLDPPLDAEIMTEEIFGPLLPIITLKKIEDSIGFIKAKPKPLVIYA
FTNDQKLKRRIVDETSSGSVTFNDSLIQYVCDTIPFGGIGESGFGSYHGKFTFDMFSHEKPVLKRSFLTEFSFRY
PPWNEQKLQLLRRLYEFDYFGFALVWLGLKRR

>XP_020241205.1 (Asparagus officinalis)

MALWKLILSILMTLSIPGSAQGSKFTKFREPYLRKASSFSSELPQVRYDYIIVGGGTAGCPLAATLSQNYSVLLL
ERGGSPYGNLNISRMQNFHISLADTSPSSPSQPFISTDGVINARARVLGGGSCINAGFYTRASTSYVKNAGW
NDELVNQSYPWIEERIVHKPKLAPWQVAVKDGLLQAGISPFNGYTYDHLYGTKVGGTIFDERGFRTTAANLL

SSGNPKNLNVLLYATVHKILFNQPKGRRPKAIGVIFKDENGQEHRAFLRRGRRSEVIVSSGTLGSPQLLMLSGI
GPKKDLKRLNIPVLIHNMHVGKGLSDNPMNSIFIPTKKPVEQSLIQTVGITKIGTFIEASSGFGQSSDSIQCHH
GIMSAEIGQLSTLPPKERTLAAAKEYARAKKDLPREAFQGGFILEKIDGPLSKGHLTLIDSDVNTTPNVTFNYF
KHPYDLERCVYGIRTIERVVRTKHIADLTNDYAYSNEVLLNMSVKANVNLIPKHTNDTVSLEQFCRDTVITIW
HYHGGCHTGKVVDRDYRVIGVNGLRVIDSSTYVNSPGTNPQATVMMMGRYMGVKMLRKRLGREAGV

>XP_010917487.1 (Elaeis guineensis)

MGALMKKEYQFLSELGLGPRNPGCYVNGSWKGSGPVVASHRPANNQVIAEVVEASMQEYEEGMQACM
DAAKIWMQIPAPKRGEIVRQIGEALRAKLQLLGRLVSLEMGKILPEGIGEVQEIIDMCDYAVGLSRQLNGSIIP
SERPNHMMMEVWNPLGIVGVITAFNFPCAVLGWNACIALVCGNCVVWKGAPTTPLITIAMTEIVAGVLEK
NNLPGAIFTAFCGGAEIGQAIACDTRIPLVSFTGSTKVGLMVQQKVNERFGKCLLELSGNNAIIVMDDADIQL
AVRSVLFAAVGTAGQRCTTCRRLLLHESIYQSVLDQLIKVYNQVKIGNPLEKGTLLGPLHTPASKENFVKGIEV
IKAQGGKILTGGSVIESEGNFVQPTIVEISPSAPVVKQELFGPVLYVMKFQTLKEAIEINNSVPQGLSSSIFTRRP
EVIFKWIGPHGSDCGIVNVNIPTNGAEIGGAFGGEKATGGGREAGSDSWKQYMRRATCTINYGSELPLAQ
GINFG

>XP_008809896.1 (Phoenix dactylifera)

MHRVALVARHVLTPSLFFRRQMSMEPGAAINRVRESGLLKTQGLIGGKWIDAYDGKTIEVQNPATGDIVTS
VAYMGKKETTDAISSAYNTFNSWSKHTASERSKCLRKWYDLMISHKEELALLMTLEQGKPLKEALGEVAYG
AGFIEFFAEEAKRIYGDIIPATLADRRLFVLKQPVGVVGAITPWNFPLAMITRKVGPALACGCTVVVKPSEYTP
LTALAAAELSLQAGIPPGVLNVVVGNASDIGDTLLQSTKVRKITFTGSTAVGKKLLAGSANTVKRISLELGGNA
PCIVFDDADLDVAVKGALAAKFRNSGQTCVCANRILVQEGIYEKFANAFAKAVQNLQVGNGLVEGIAQGPL
INEAAVEKVEKFIKDATSKGANVILGGRRHSLGMTFYEPTVLGNVNDEMLVSREEVFGPVAPLLRFKTEEDAI
HIANDTNAGLAAYIFTTSIPRSWRVTEALEYGLVGVNEGLISTEVAPFGGFKQSGLGREGSKYGIDEYLELKYV
CLGNMKEAYTK

>XP_020098233.1 (Ananas comosus)

MNRFISYRELGAGARRAAWSWFSPSISLASRSVHSPSFATVDVEDLSSSHPAEVKNLVQGNWKTSANWSSI
LDPLNGDRFIKVAEVQETEIKPLVKSLSKCPKYGLHNPLRAQDRYVMYGDISAKAANMLAQPEVSHFFTRLIQ
RVSPKSYKQALAEVNVSQKFLENFSGDQVRFLARSFAVPGNHLGQHSHGYRWPYGPVAIITPFNFPLEIPLL
QVMGALYMGNKPVLKVDSKVSIVMEQMLRLLHDCGLPMEDVDFINSDGITMNKLLIEAKPQMTLFTGSSH
VAEKLASDLNGRIKLEDAGFDWKILGPDVQEVDYVAWVCDQDAYACSGQKCSAQSILFVHENWASTGLID
KLQCLAGKRKLEDLTIGPVLTVTTASMLEHIHKLLKIPGSKILFGGEPLENHSIPEVYGALKPTALFVPLGEMLKT
DNFELVTREIFGPFQIITEYRHDQLELVLDACERMHAHLTAAVVSNDPLFLQDVIGRSVNGTTYAGIRARTTG
APQNHWFGPAGDPRGAGIGTPEAIKLVWSCHREVIYDIGPLPKDWEVPAAT

>XP_020265044.1 (Asparagus officinalis)

MAFWWPLLVAGIAFAICKLLFMLIPPNVPSIEVDASDVLEDGSQTKENSYIYIPRKGKSAQTDKVQCYEPAT
MKYLGYFPALTPDEVKEHVAQARKAQKTWAKSSFRQRRQFLRILLKYIIEHQELICEISSRDTGKTMVDASLG
EIMTTCEKITWLLDEGEKWLKPEYRCSGRSMLHKRAKVEFYPLGVIGAIVSWNYPFHNIFNPMLAAVFSGN
AAVIKVSEHASWSGCFYLRIIQAALAAVGAPDNLVHVITGFAETGQALVSSVDKIIFVGSPGVGKMIMRKASE
TLIPVTLELGGKDAFIVCEDVDVPHVVQIATRAALQSSGQNCAGAERFYVHKDVYSTFVAQIVKLVILMVHG
PPSAGKYDMGAICMQEHADKLQNLVNDALEKGAEIAGRGSFGHLGEDAVDQFFPPTVLVNVNHTMKLM
QEEAFGPIIPIMKFSDDEEVIKLANDSNYGLGCAVFSGSQKRAIAIASQLHCGVAAINDFASTYMCQSLPFGG

VKHSGFGRFAGVEGLRSCCLVKSVVEDRFWPYIKTVIPKPIQYPIAENGFEFQESLVELLYGMNVWDRLRAV
VNVLKIITEKNGTPITEKKRRN

> >AIX10972.1 (Ornithogalum longebracteatum)

MAFSTALRSAAPLVDGSRDLFPFSDSTIRASDSFKVSCHSTKSSLRSASVSSSIVGASISPLEVVPSQISSGRVIQ
PVKATATEAPPAVARSSSGGKTKIGINGFGRIGRLVLRIATSRDDVEVVAVNDPFIDAKYMAYMLKYDSTHG
LFDGTIKVVDQSALEINGKNVVVTSKRDPAEIPWGDFGAEYVVESSGVFTTVDKASSHLKGGAKKVVISAPS
ADAPMFVVGVNEKKYEPNMNVVSNASCTTNCLAPLAKVVHEEFGIVEGLMTTVHATTATQKTVDGPSMK
DWRGGRGAGQNIIPSSTGAAKAVGKVLPELNGKLTGMAFRVPTPNVSVVDLTCRLEKSASYDDVKAAIKFA
SDGPLRGILGYTDEDVVSNDFIGDSRSSIFDAKAGIGLSASFMKLVSWYDNEWGYSNRVLDLIEHMALVSAT
R

>CsADH54788 (Crocus sativus)

MAFDGEKAMEMVKELRESFNKGTTRGYEWRVKQLKAMEKMIEEKEKDIMDALESDLSKPQLESFLHEVS
MAKSACQFAAKNLKRWMKPEKVPAQITTFPSVGNIVAEPFGVVLIISAWNYPFLLSLDPVIGAIASGNTVVL
KPSEIAPATSALFARILPDYVDTSCVRVVEGAVPETTALLEQKWDKIFYTGNGKVGRVVMAAAAKHLTPVVL
ELGGKSPVVVDSNIDLKVATKRIVVGKWGCNNGQACIAPDYIVTTKSFAPKLVESLKITLERFYGKDPLETADL
SRIVNSNHVARLTRLLDDDMVSGTIVYGGQRDEKRLKIAPTLLLDVPEDSLIMKEEIFGPLLPIITVDKIEDSFAV
INSKPKPLAAYLFTKNKNLERMFVETVSSGGMLINDTALHLANPYLPFGGVGESGTGSYHGKFSFDAFSHKK
AVLSRGFGGELGARYPPYTNKKQKILRALLAGNIIALVWALLGFSKS

>CsADH3898 (Crocus sativus)

MLQSPLRRASTLRTWRYPASLHRLSCFSASPQASPLHGNPTSRVPNFIGGQFLESRSESSINVINPATQEIVSR
VPLTTNEEFKAAVGAAKCAFPKWRNTPITARQRVMFKLQELIRRDMDKLALNITTEQGKTLKDAQGDVFRG
LEVVEHACGMGTLQMGEYVSNVSHGIDTYSIREPLGVCAGICPFNFPAMIPLWMFPVAVTCGNTFVLKPSE
KDPGASMLLAELAMEAGLPDGVLNIVHGANDTVNNICDDEDIRAVSFVGSNTAGMHIYSRAAAKGKRVQS
NMGAKNHAIIMPDASPDATINALVAAGFGAAGQRCMALSTAVFVGGSKSWEDEIIKHASALKVNAGVEPE
TDLGPVISRQAKDRICRLVQSGIDSGARVVLDGRNIVVPGYKDGNFVGPTLLADVSSDMECYKEEIFGPVLLC
MQADSLEDAIDIVNRNKYGNGASIFTTSGVSARKFQSEIEAGQVGINVPIPVPLPFFSFTGSKASFAGDLNFY
GKAGIQFFTQIKTVTQQWKDLPSQGVSLAMPTSQKS

>CsADH-20158 (Crocus sativus)

MAAAKKIASILLSRSSSASRSFHLRRGAASGIQRFATAAVVEEEPITPPVQINHTQHLINGKFVDSASGKTFPTF
DPRTGEVITHVSQGEVEDVNRAVAAARKAFDEGPWPKMTAYERSRILFRAADLIEKHNDEIAALESWDSGK
TYEQSSLVEIPMVIRYMRYYAGWADKIHGLVVPADGPHHVQVLYEPIGVAGQIIPWNFPLVMFAWKVGPA
LACGNTVIIKTAQQTPLSALYVAKLFHEAGLPDGVLNVVSGYGPTAGAALASHMDVDKLAFTGSTPTGKVV
MELAAKSNLKSVILELGGKSPMIVLEDGDVDQAVELAHSALFFNQGQCCCAGSRTFVHERVYDEFVEKSKA
RALKRVVGDPFRKGVEQGPQIDGDQFNRILHYIKSGIDGGASLVTGGDRLGSKGFYIQPTIFSDVKDDMIIAK
EEIFGPVQSIFKFSDLDEVIRRSNASPFGLAAGVFTNNMDNANTLMRALRVGTVWINCYGVFDAALPFGGY
KMSGQGREKGIDSLKAYLEVKSVVTTLKNPAWL

>CsADH-11367 (Crocus sativus)

MAAAKRIASSLIARSLSTSSRSFLLRRGDAALGIQRFGTAAAFEEQPIKPSVQINHTQLLINGNFVDSASGKTFP
TLDPRTGEVIAHVAEGDAEDVNRAVAAARKAFDEGPWPRMTAYERSRILLRAADLIEKHNDEISALESWDS
GKPYEQSALAEIPLVVRLMRYYAGWADKIHGLVVPADGPHHVQVLHEPIGVAGQIIPWNFPLVMFAWKV
GPALACGNAIVIKTAEQTPLSALYVARLFHEAGLPDGVLNVISGFGPTAGAALASHMDVDKLAFTGSTATGK
VVLELAARSNLKPVTLELGGKSPMIILEDADIDNAVELAHFALFFNQGQCCCAGSRTFVHERIYDEFVEKSKA
RALRRVVGDPFRKGVEQGPQIDNEQFNKILRYIKLGKDSGASLVTGGDRLGSKGFYIQPTIFSDVQDDMVIA
QDEIFGPVQTILKFNNLDEVIRRANASRYGLAAGLFTNNMDHANTLMRALKVGTIWINCFDVFDAALPFGG
FKMSGQGREKGIDSLKAYLQVKSVVTALKNPAWL
